# Orexin System: The Key for a Healthy Life

**DOI:** 10.3389/fphys.2017.00357

**Published:** 2017-05-31

**Authors:** Sergio Chieffi, Marco Carotenuto, Vincenzo Monda, Anna Valenzano, Ines Villano, Francesco Precenzano, Domenico Tafuri, Monica Salerno, Nicola Filippi, Francesco Nuccio, Maria Ruberto, Vincenzo De Luca, Luigi Cipolloni, Giuseppe Cibelli, Maria P. Mollica, Diego Iacono, Ersilia Nigro, Marcellino Monda, Giovanni Messina, Antonietta Messina

**Affiliations:** ^1^Department of Experimental Medicine, Section of Human Physiology and Unit of Dietetic and Sport Medicine, Università degli Studi della Campania “Luigi Vanvitelli”Naples, Italy; ^2^Department of Mental Health, Physical and Preventive Medicine, Clinic of Child and Adolescent Neuropsychiatry, Università degli Studi della Campania “Luigi Vanvitelli”Naples, Italy; ^3^Department of Clinical and Experimental Medicine, University of FoggiaFoggia, Italy; ^4^Department of Motor Sciences and Wellness, University of Naples “Parthenope”Naples, Italy; ^5^Department of Medical-Surgical and Dental Specialties, Università degli Studi della Campania “Luigi Vanvitelli”Naples, Italy; ^6^Department of Psychiatry, University of TorontoToronto, ON, Canada; ^7^Department of Anatomical, Histological, Forensic and Orthopaedic Sciences, Università degli Studi di Roma La SapienzaRome, Italy; ^8^Department of Biology Università degli Studi di Napoli Federico IINaples, Italy; ^9^Neurodevelopmental Research Lab, Biomedical Research Institute of New JerseyMorristown, NJ, United States; ^10^Neuroscience Research, MidAtlantic Neonatology Associates, Atlantic Health SystemMorristown, NJ, United States; ^11^Neuropathology Research, MANA/Biomedical Research Institute of New JerseyMorristown, NJ, United States; ^12^CEINGE-Biotecnologie Avanzate ScarlNaples, Italy

**Keywords:** orexin, obesity, emotional stress, narcolepsy

## Abstract

The orexin-A/hypocretin-1 and orexin-B/hypocretin-2 are neuropeptides synthesized by a cluster of neurons in the lateral hypothalamus and perifornical area. Orexin neurons receive a variety of signals related to environmental, physiological and emotional stimuli, and project broadly to the entire CNS. Orexin neurons are “multi-tasking” neurons regulating a set of vital body functions, including sleep/wake states, feeding behavior, energy homeostasis, reward systems, cognition and mood. Furthermore, a dysfunction of orexinergic system may underlie different pathological conditions. A selective loss orexin neurons was found in narcolepsia, supporting the crucial role of orexins in maintaining wakefulness. In animal models, orexin deficiency lead to obesity even if the consume of calories is lower than wildtype counterpart. Reduced physical activity appears the main cause of weight gain in these models resulting in energy imbalance. Orexin signaling promotes obesity resistance via enhanced spontaneous physical activity and energy expenditure regulation and the deficiency/dysfunction in orexins system lead to obesity in animal models despite of lower calories intake than wildtype associated with reduced physical activity. Interestingly, orexinergic neurons show connections to regions involved in cognition and mood regulation, including hippocampus. Orexins enhance hippocampal neurogenesis and improve spatial learning and memory abilities, and mood. Conversely, orexin deficiency results in learning and memory deficits, and depression.

## Introduction

Orexin A and B are excitatory hypothalamic neuropeptides playing a relevant role in different physiologic functions such as sleep/wake rhythms and thermoregulation, control of energy metabolism, cardiovascular responses, feeding behavior, and spontaneous physical activity (SPA).

At the end of the last century, Sakurai et al. (1998) firstly described Hypocretins 1 and 2 as regulators of feeding and appetite behavior, produced in a specific hypothalamic region (Edwards et al., 1999; Haynes et al., 2000, 2002).

More recent studies focused the attention on the role of the orexins in mood and emotional regulation, energetic homeostasis, reward mechanisms, drug addiction, arousal system, and sleep and wakefulness (Peyron et al., 2000; Thannickal et al., 2000; Hara et al., 2001; Yamanaka et al., 2003a; Harris et al., 2005; Narita et al., 2006). However, the function of orexins in metabolism pathways are far to be completely understood.

## The orexin/hypocretin system

**Figure 1:**
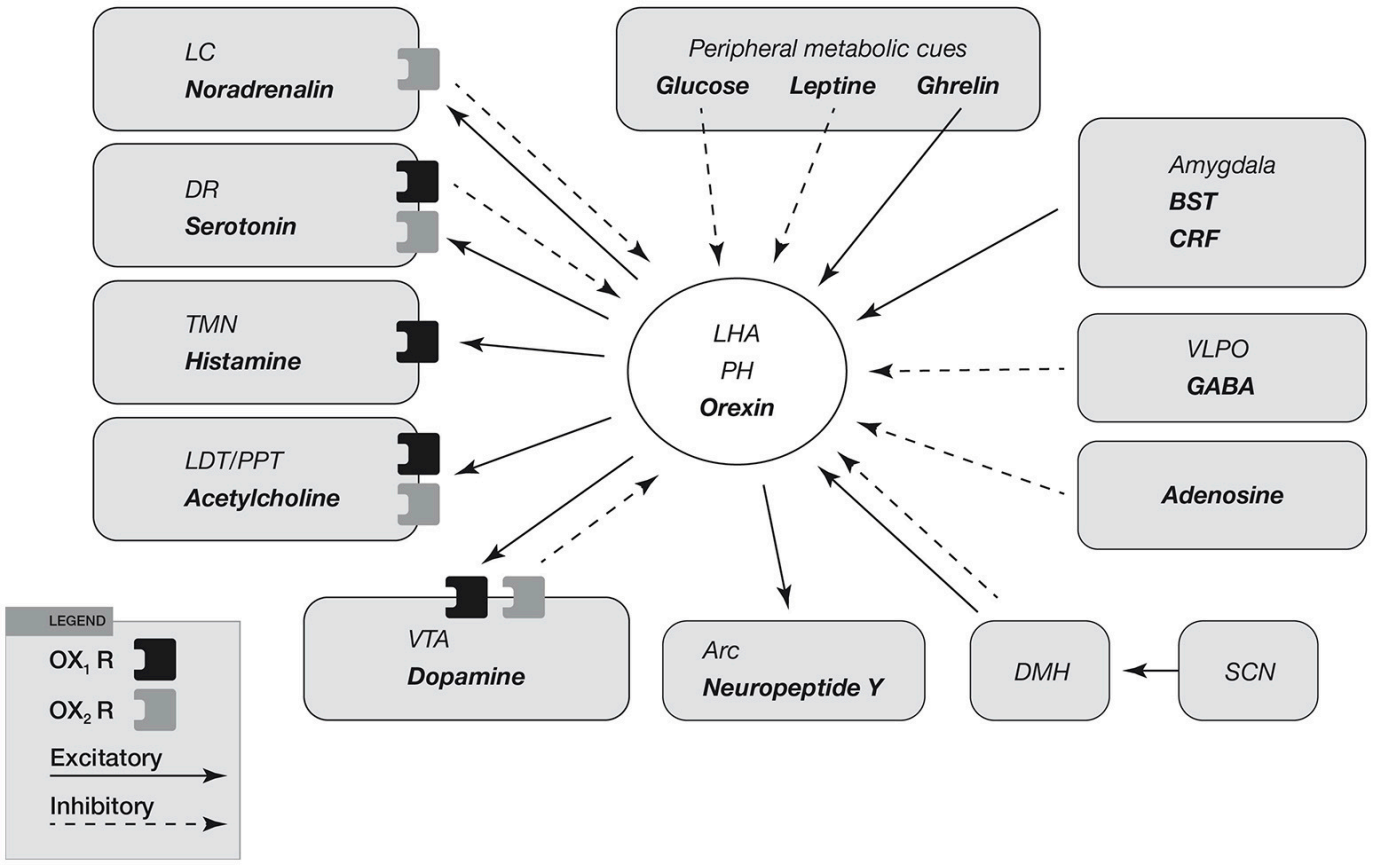
Schematic representation of orexin system. Orexin A and orexin B are derived from a common precursor peptide, prepro-orexin. The actions of orexins are mediated via two G protein-coupled receptors named orexin-1 (OX_1_R) and orexin-2 (OX_2_R) receptors. OX_1_R is selective for orexin A, whereas OX_2_R is a non-selective receptor for both orexin A and orexin B. OX_1_R is coupled exclusively to the Gqsubclass of heterotrimeric G proteins, whereas OX_2_R couples to Gi/oand/or Gq.

In Mammals, orexin A and orexin B are synthesized in the lateral hypothalamic and perifornical areas (Peyron et al., 1998; Nambu et al., 1999), starting from a common polypeptide precursor (prepro-orexin) through proteolytic processing (Figure 1).

**Figure 2:**
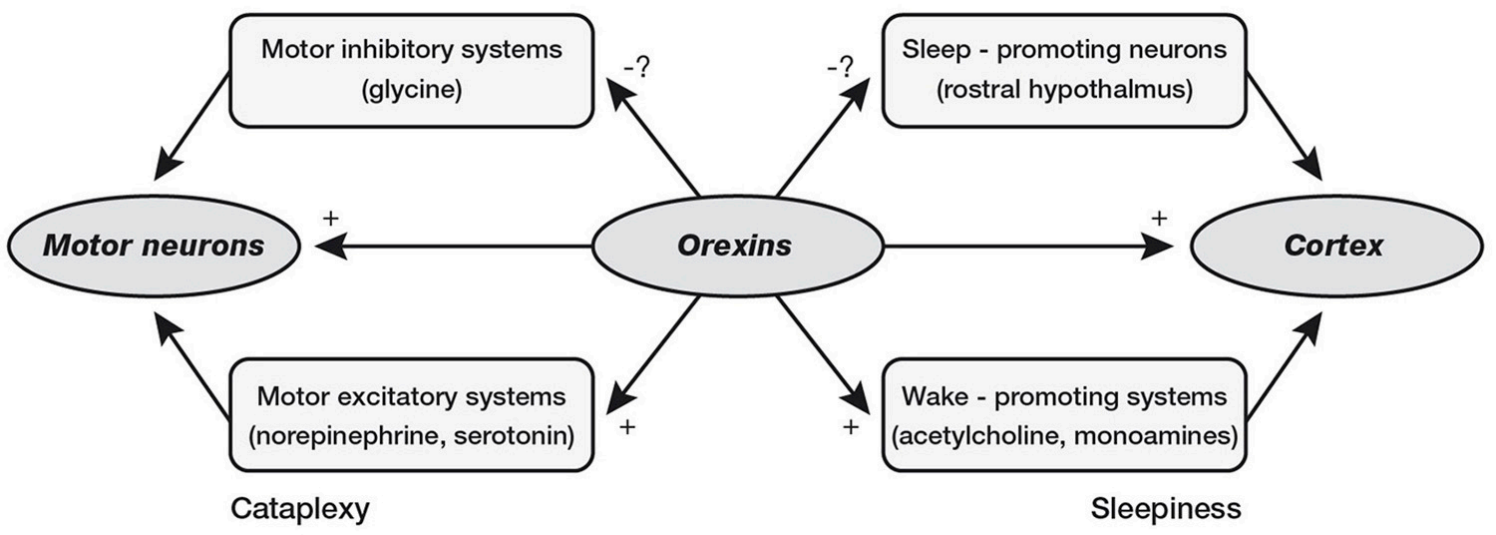
Schematic drawing showing main projections of orexin neurons. This figure summarizes predicted orexinergic projections in the human brain. Please note that distributions of orexin fibers and receptors (OX_1_R, OX_2_R) are predicted from the results of studies on rodent brains since most histological studies on the orexin system have been carried out in mice and rats. Circles show regions with strong receptor expression and dense orexinergic projections. Orexin neurons originating in the lateral hypothalamic area (LHA) and posterior hypothalamus (PH) regulate sleep and wakefulness and the maintenance of arousal by sending excitatory projections to the entire CNS, excluding the cerebellum, with particularly dense projections to monoaminergic, and cholinergic nuclei in the brain stem and hypothalamic regions including the locus coeruleus (LC, containing noradrenaline), tuberomammillary nucleus (TMN, containing histamine), raphe nuclei (Raphe, containing serotonin), and laterodorsal/pedunclopontine tegmental nuclei (LDT/PPT), containing acetylcholine). Orexin neurons also have links with the reward system through the ventral tegmental area (VTA, containing dopamine) and with the hypothalamic nuclei that stimulate feeding behavior.

Orexin A is a neuropeptide composed of 33 amino acids with an amino(N)-terminal pyroglutamyl residue, two intra-chain disulphide bonds and carboxy (C)-terminal amidation, while Orexin B is a linear neuropeptide sized 28 amino acids, C-terminally amidated. The N-terminal portion presents more variability, whilst the C-terminal portion is similar between the two subtypes. The orexins activity is modulated by their specific receptors (OX_1_R, OX_2_R). OX_1_R presents higher affinity for orexin A than B and transmits signals throughout the G-protein class activating a cascade that leads to an increase in intracellular calcium concentration. By contrast, OX_2_R binds the two subtypes of orexin with similar affinities, probably associated to a G inhibitory protein class (Xu et al., 2013). These differences seem to suggest different physiological roles for OX_1_R and OX_2_R (Trivedi et al., 1998). Different physiological roles of OX1R and OX2R seem to be supported by the observation that mRNAs receptors show complementary distribution patterns (Figure 2):

- OX_1_R is distributed in prefrontal and infralimbic cortex (IL), hippocampus (CA2), amygdala, stria terminalis bed nucleus (BST), PVT, anterior hypothalamus, dorsal raphe (DR), ventral tegmental area (VTA), locus coeruleus (LC), and laterodorsal tegmental nucleus (LDT)/pedunculopontine nucleus (PPT) (Trivedi et al., 1998; Lu et al., 2000);

- OX_2_R is distributed in amygdala, TMN, Arc, dorsomedial hypothalamic nucleus (DMH), paraventricular nucleus (PVN), LHA, BST, PVT, DR, VTA, LDT/PPT, CA3 in the hippocampus, and medial septal nucleus (Lu et al., 2000).

## Modulation of orexin neurons

Electrophysiological studies on transgenic mice have identified several neurotransmitters and neuromodulators influencing the activation or inhibition in orexin neurons activity. Specifically, GABA (Xie et al., 2006), noradrenaline and serotonin seem to inhibit the activity of orexin neurons (Yamanaka et al., 2003b), as dopamine acts through activation of α2-adrenoceptors (Yamanaka et al., 2006). Moreover, agonists of ionotropic glutamate receptors tend to excite orexin neurons, while glutamate antagonists inhibit their activity (Li et al., 2002) indicating that glutamatergic neurons can tonically activate orexin neurons. Cholecystokinin, neurotensin, oxytocin, and vasopressin enhance orexin neurons activity (Tsujino et al., 2005; Tsunematsu et al., 2008) by modulation of adenosine and CO_2_ concentrations (Liu and Gao, 2007; Williams et al., 2007).

## Sleep/wake regulation

Interestingly, orexin system seems to be crucial for maintenance of wakefulness state, as demonstrated by narcolepsy caused by orexin deficiency in Human and Animals (Chemelli et al., 1999; Lin et al., 1999). Narcolepsy is a neurological disease affecting ~1 in 2,000 individuals in the United States (Mignot, 1998), characterized by chronic daytime sleepiness, sleep attacks, and possibility of cataplexy, hypnagogic hallucinations and sleep paralysis. These symptoms are not necessary to be present all together and narcolepsy may be identified and diagnosed by standard polysomnography (PSG) at all ages, including childhood. Narcolepsy is the results of orexin-containing neurons loss, which tend to increase their activity during wakefulness activating aminergic nuclei such as locus coeruleus, raphe nuclei, and tuberomamillary nucleus with maintaining wake state and preventing of inappropriate transitions into sleep, particularly REM sleep phases (Saper et al., 2001; España and Scammell, 2011). Narcolepsy is usually classified into two subtypes: (a) Narcolepsy with cataplexy (type 1); and (b) Narcolepsy without cataplexy (type 2). On the other hand, the orexin in sleep/wake regulation and pathophysiology of narcolepsy may be not limited to the activation/deactivation of cataplexic phenomena and/or sleep attacks. In fact, affected children and adolescents present specific cognitive impairments (Posar et al., 2014), which pinpoint the close relationship between sleep and cognition (Esposito and Carotenuto, 2014). Moreover, probably due to the same role in sleep modulation, orexin seems to be also involved in the pathogenesis of migraine (Rainero et al., 2008) as suggested by the link between NREM sleep instability and risk of cognitive impairments and behavioral problems (McCoy and Strecker, 2011; Bruni et al., 2012; Colonna et al., 2015; Carotenuto et al., 2016, Figure 3).

**Figure 3:**
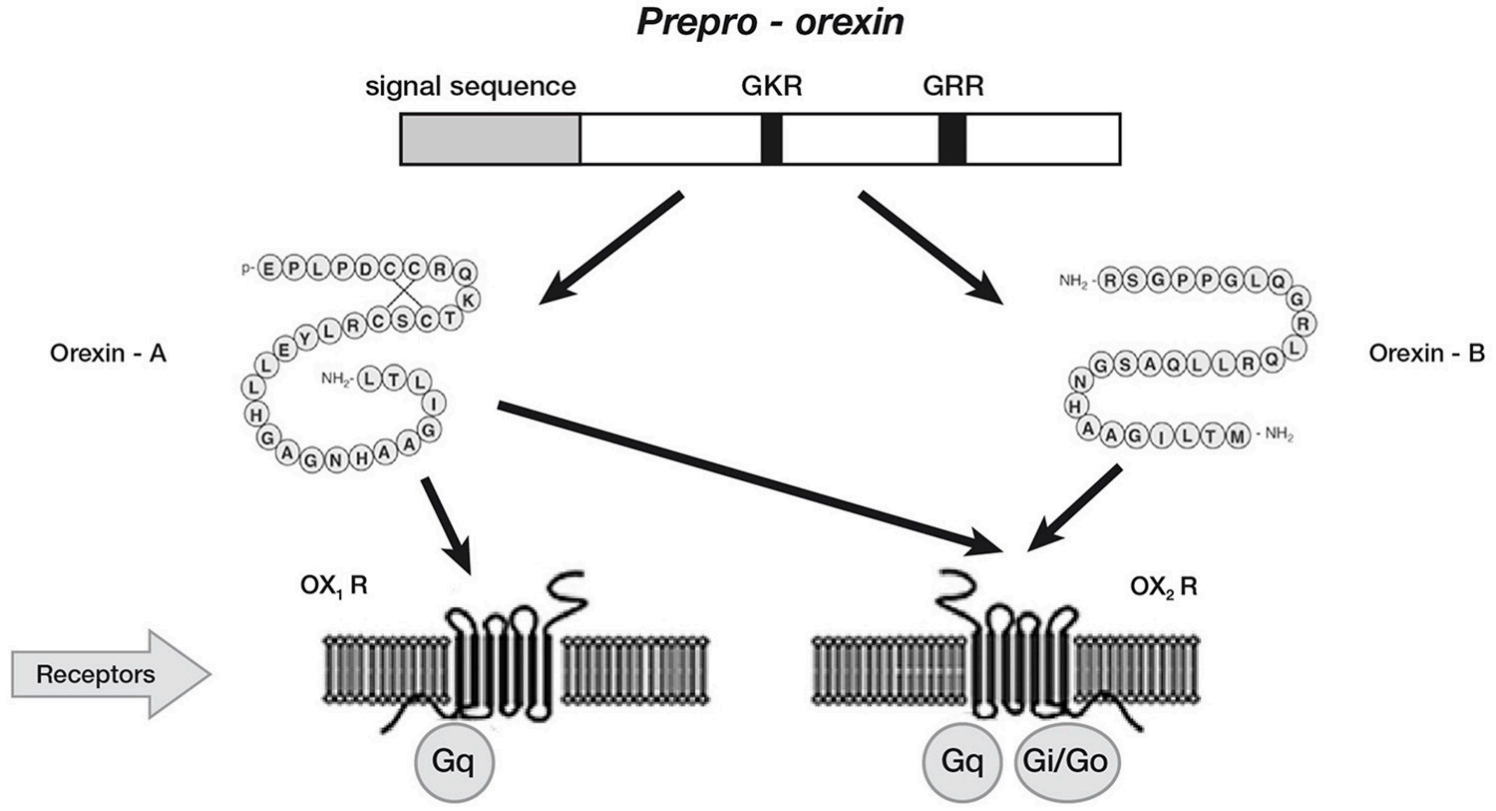
Loss of orexin signaling could cause cataplexy by reducing activity in motor excitatory systems of brainstem or by providing less suppression of the motor inhibitory systems. Loss of orexin could cause sleepiness by reducing activity in the cholinergic and monoaminergic arousal systems or by reducing inhibition of sleep-promoting neurons in the rostral hypothalamus (preoptic area).

## Feeding behaviors and energy homeostasis

Orexin A seems to regulate feeding behaviors and energy expenditure as evidenced by the intracerebroventricular (icv) injection of orexins effects during the light period, which induces feeding behavior in rodents and zebrafishes, probably for a direct action on lateral hypothalamic area containing neurons modulated by glucose concentration. In fact, the high concentrations of glucose and leptin tend to hyperpolarize orexinergic neurons, while low concentrations of glucose and ghrelin depolarize them. Therefore, the orexinergic system discriminate physiological variation in glucose levels due to meals modulating in this way energy balance according to food intake (Monda et al., 2008; Messina et al., 2014). Moreover, transgenic mice with gradual and then loss of hypothalamic orexin-containing neurons show feeding abnormalities and dysregulation in energy homeostasis determining obesity despite the reduction of food intake/calories. Interestingly, it has been reported an increased prevalence of obesity in narcoleptic subjects in all ages (Yokobori et al., 2011).

Shiuchi et al. (2009) observed that the regulation mediated by orexinergic system on muscle glucose metabolism is due to activation of β_2_-adrenergic signaling and consequently peripheral energy expenditure. A persistent wake-state mediated by orexins could also be important for food intake motivation. When facing reduced food availability, animals adapt with a longer awake period, revolutionizing their normal pattern of activity (Viggiano et al., 2009). During starvation the activation of orexin neurons mediated by low leptin and glucose levels, might modulate their activity according to energy expenditure and stores to maintain wakefulness, whilst orexin neuron-ablated mice fail to respond to fasting with increased wakefulness and activity (Viggiano et al., 2010; Messina et al., 2013; Chieffi et al., 2017; Villano et al., 2017). These data confirm that orexin neurons mediate energy balance and arousal, maintaining a consolidated state of wakefulness in hungry animals in order to promote alertness.

## The role of orexin system in obesity

Obesity is a complex multifactorial condition lowering health quality and many effects such as metabolic syndrome, type 2 diabetes mellitus, coronary heart disease, sleep apnea syndrome, and reduced in life expectancy (Must et al., 1999). In the last decades, the incidence of obesity has increased both in children and adults worldwide (Flegal et al., 2010). Environmental and genetic factors cause large variations among human susceptibility to obesity. Physical activity and the so called “non-exercise induced thermogenesis” (NEAT) are factors determining this variability and susceptibility. The term NEAT includes all types of energy expenditure not associated with formal exercise, such as standing and fidgeting (Levine, 2002). A complementary concept to that of NEAT is the spontaneous physical activity (SPA) describing any type of physical activity not qualified as voluntary exercise. Together NEAT and SPA are hereditable but not interchangeable, because NEAT refers to energy expenditure while SPA describes the types of physical activity resulting in NEAT. Therefore, SPA induces an important variability in sensitivity to obese subject that spend less time standing than leans (Levine et al., 2005). Orexin signaling would promote obesity resistance via enhanced SPA and energy expenditure regulation and the deficiency/dysfunction in orexins system lead to obesity in animal models despite of lower calories intake than wildtype associated with reduced physical activity. On the other hand, the body weight regulation seems to be complex according to the lack of orexin neurons. In 2012, Perez-Leighton et al. (2012) highlighted the protective role of intratecal administration orexin A against obesity in mice models.

Orexin A has also been discovered to promote SPA and NEAT as effect of administration into specific cerebral areas (i.e., rostral LH, hypothalamic paraventricular nucleus, nucleus accumbens, locus coeruleus, dorsal raphe nucleus, tuberomamillary nucleus, substantia nigra, Hara et al., 2005). In this light, orexinergic neurotransmission may be an interesting and new pharmacologic target for obesity therapy (Zink et al., 2014). Low levels of orexin in CNS and peripheral tissues were found in animal models of obesity diet-induced (Hara et al., 2005), and adipose tissue in obese humans subjects showed lower concentrations of orexin and reduced in its receptors activity (Hara et al., 2005). A study conducted by Levin et al. (1997) on Sprague Dawley rats showed that models fed with a high-fat diet gained no more weight than chow-fed controls. Obesity prone (OP) and obesity resistant (OR) models present different profiles in weight gaining despite of no differences in energy intake (Levin et al., 1997; Messina et al., 2015; Messina A. et al., 2016). The OR group showed lower body weight and fat mass on a low-fat diet and gain less weight when fed with high-fat diet than OP group. Furthermore, OR rats lean group suggest that the negative caloric benefit of OXA-induced SPA appears to outweigh the positive calories due to OXA-induced hyperphagia (Levin et al., 1997; Carotenuto et al., 2010; Esposito et al., 2013). OXA action on SPA had a longer duration when compared with that above food intake (Carotenuto et al., 2011; Bellini et al., 2013); OR rats have higher endogenous SPA thus reflecting their higher sensitivity to SPA- promoting stimuli such as lower caloric intake. By contrast OP rats displayed lower SPA endogenous levels after a high-fat diet administration if compared to their OR group counterpart (Levin et al., 1997; Esposito et al., 2012a). Conversely, obesity tends to increase also the prevalence of migraine in all ages of life (Esposito et al., 2012b; Verrotti et al., 2012, 2015).

## Reward system

Orexin system seems to play a unified role in coordinating motivational activation under numerous behavioral conditions (James et al., 2016) as showed, for example, by its involving in alcohol use and drug-addiction (Martin-Fardon et al., 2016; Walker and Lawrence, 2016). Recent studies focused their attention on reward system modulation by orexin system. Treating narcoleptic patients with amphetamine-like drugs (Di Bernardo et al., 2014) did not lead to addiction to these drugs (Monda et al., 2014). Wild-type mice are more susceptible to developing morphine dependence in comparison with orexin knockout mice (Chieffi et al., 2014b). Furthermore, reward brain circuits in humans affected by narcolepsy were identified as abnormal (Viggiano et al., 2014) However, the mismatch between predicted reward and reward subsequently received was significantly higher in Parkinson's disease (PD) compared to narcoleptic, independent of reward magnitude and valence as showed by cataplexy that may be triggered by both positive and negative emotions (Mensen et al., 2015).

**Figure 4:**
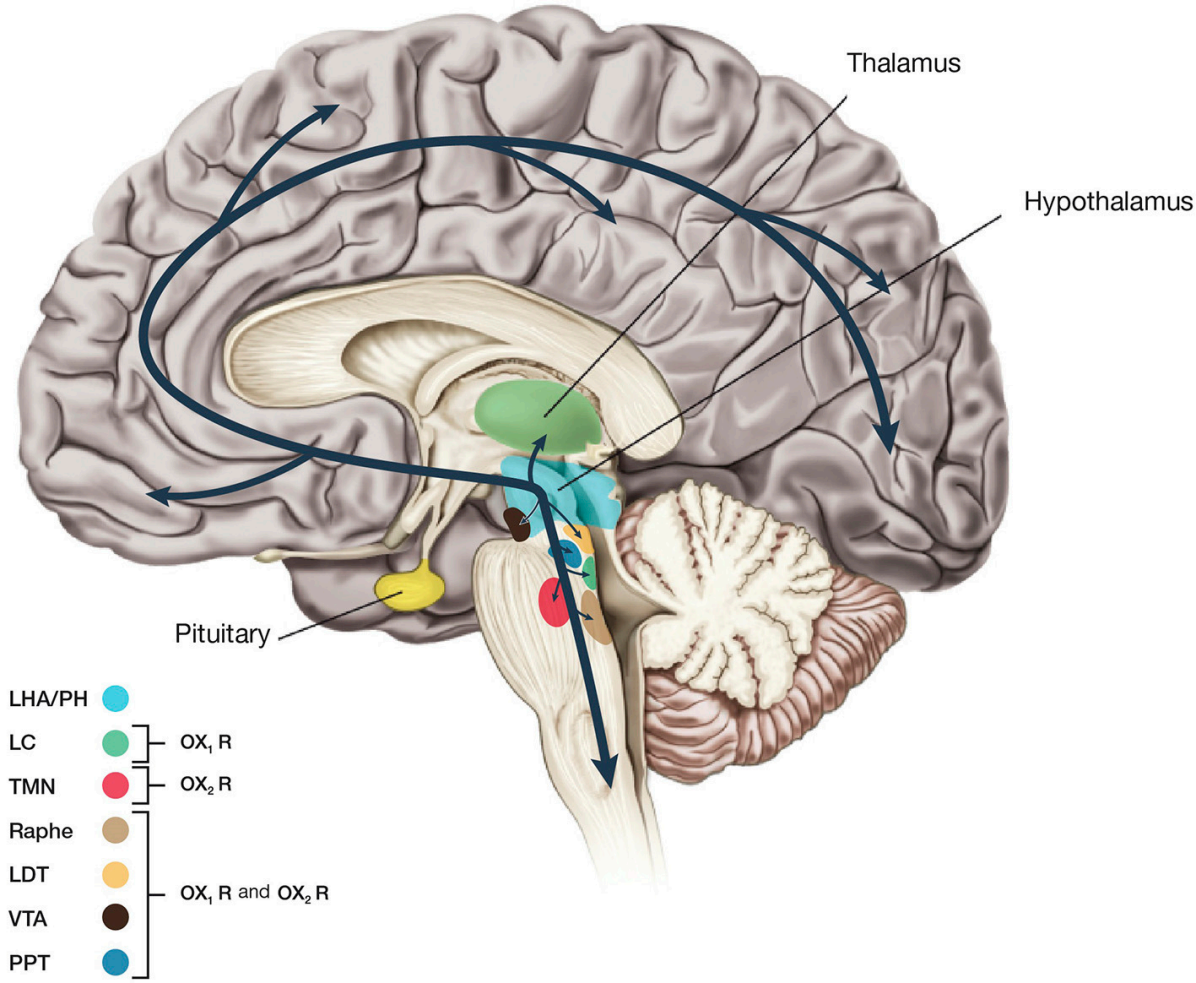
Input and output of orexin neurons at interface of sleep, stress, reward, and energy homeostasis. Orexin neurons in the lateral hypothalamic area (LHA) and posterior hypothalamus (PH) are placed to provide a link among limbic system, energy homeostasis, brainstem and other systems. Arrows show excitatory projections and broken arrows inhibitory projections. Gray semicircles indicate OX1R and black semicircles indicate OX2R. Neurotransmitters/modulators are underlined. LC, DR, and TMN are wake-active regions, VLPO is sleep-active region, and LDT/PPT is REM-active region. Orexin neurons promote wakefulness through monoaminergic nuclei that are wake-active. Stimulation of dopaminergic centers by orexins modulates reward systems (VTA). Peripheral metabolic signals influence orexin neuronal activity to coordinate arousal and energy homeostasis. Stimulation of neuropeptide Y neurons by orexin increases food intake. The SCN, the central body clock, sends input to orexin neurons via the DMH. Input from the limbic system (amygdala and BST) might be important to regulate the activity of orexin neurons upon emotional stimuli to evoke emotional arousal or fear-related responses. Abbreviations: BST, bed nucleus of the stria terminalis; VLPO, ventrolateral preoptic area; LC, locus ceruleus; DR, dorsal raphe; TMN, tuberomammillary nucleus; LDT, laterodorsal tegmental nucleus; PPT, pedunculopontine tegmental nucleus; VTA, ventral tegmental area; SCN, suprachiasmatic nucleus; DMH, dorsomedial hypothalamus; Arc, arcuate nucleus.

Regulatory mechanisms at the base of reward system are shown in Figure 4. It seems to be clear that orexin neurons modulate reward system and play a predominant role in mechanisms of drug addiction. Many reports suggest a critical role of orexin signaling in neural plastic effects at glutamatergic synapses in the ventral tegmental area (VTA) (Figure 4).

## Links with brain

Animal studies suggest that orexinergic system may enhance hippocampal neurogenesis influencing learning and memory processes. In 2004, Wayner et al. (2004) reported that local dentate gyrus perfusion with orexin-A in anesthetized rats enhanced long-term potentiation (LTP) strengthen the link between structural and functional hippocampal plasticity. Moreover, Wayner et al. (2004) showed that LTP enhancement may be blocked in SB-334867 pre-treated rats SB-334867, a specific Ox1R antagonist (Wayner et al., 2004). The effects of dentate gyrus-OX1Rs antagonization on LTP occurred also in freely moving rats impairing spatial memory in Morris water maze (Wayner et al., 2004). Some studies examined the effects of the administration of orexin-A in rats treated with Pentylenetetrazol (PTZ) (Zhao et al., 2014). PTZ induces seizures resulting in the hippocampal atrophy, learning and memory deficits and decrease of cerebrospinal fluid-level of orexin-A (Coppola et al., 2010), while the intracerebroventricular injection of orexin-A in PTZ-kindled rats tend to attenuate these impairments, enhancing neurogenesis in the dentate gyrus (Zhao et al., 2014). Interestingly, in rats treated with orexin-A more than 50% of newborn cells differentiated into neurons, whereas only approximately 30% of the newborn cells differentiated into neurons in the control group (Zhao et al., 2014).

Moreover, Orexin-A seems to be implicated in social memory, the ability to distinguish and remember familiar from novel conspecifics (Yang et al., 2013). Yang et al. (2013) reported that orexin/ataxin-3-transgenic (AT) mice, in which orexin neurons degenerate by 3 months of age, displayed deficits in long-term social memory. Nasal administration of exogenous orexin-A restored social memory and enhanced synaptic plasticity in the hippocampus (Yang et al., 2013). A decrease of Orexin-A was found in animal models of depression and its intracerebroventricular administration reduced depression symptoms and increases the number of cells in the dentate gyrus (Arendt et al., 2013). Then, it is possible that the enhancement of cell proliferation in the dentate gyrus by orexin-A might have an antidepressive-like effect.

Interestingly, physical exercise produces an increase of orexin-A level in cerebrospinal fluid of rats, dogs and cats, and in plasma of humans (Messina G. et al., 2016). Note that the orexin-A rapidly cross the blood-brain barrier, probably by simple diffusion, having a high degree of lipophilicity. Furthermore, physical exercise is (a) an effective tool for enhancing cognitive performance and regulating mood and (b) produced morphological and functional changes of brain regions that play central roles in successful everyday functioning, such as frontal and temporal cortices, and the hippocampus located in the inner (medial) region of the temporal lobe. The frontal lobe is important for cognitive function (Iavarone et al., 2007; Chieffi et al., 2009, 2012; Boscia et al., 2015), the temporal lobe for memory function (Chieffi et al., 2004, 2014a, 2015; Marra et al., 2013; Franco et al., 2014). The factors most likely involved in exercise-induced hippocampal neurogenesis are the microcirculation and the production of neurotrophic factors such as the BDNF, VEGF, and IGF-1 (Cotman et al., 2007). Another putative factor that might contribute to the beneficial effects of exercise is the orexin-A.

## Concluding remarks

Orexin is necessary for healthy life because of the important and relevant homeostatic functions controlled and organized directly or not. Orexin role is not only limited to the brain areas, because it involved also in metabolic regulation.

## Author contributions

AM, VM, AV, IV, MR: conceived the study, participated in its design, and wrote the manuscript. FP, DT, MS, NF, FN, LC, EN: contributed to the conception and design. SC, MC, VD, GC, MPM, DI, MM, GM: drafted the article and revised it critically for important intellectual content. DI revised grammar and English form; GM: final approval of the version to be published. All authors read and approved the final manuscript.

### Conflict of interest statement

The authors declare that the research was conducted in the absence of any commercial or financial relationships that could be construed as a potential conflict of interest. The reviewer IS declared a shared affiliation, though no other collaboration, with several of the authors MC, FP, MR to the handling Editor, who ensured that the process nevertheless met the standards of a fair and objective review.

## Abbreviations

SPA: spontaneous physical activity
OX_1_R: orexin receptor 1
OX_2_R: orexin receptor 2
IL: infralimbic cortex
CA2: hippocampus CA2 area
BST: stria terminalis bed nucleus
PVT: paraventricular thalamus
DR: dorsal raphe
VTA: ventral tegmental area
LC: locus coeruleus
LDT: laterodorsal tegmental nucleus
PPT: pedunculopontine nucleus
TMN: tuberomammillary nucleus
Arc: arcuate nucleus
DMH: dorsomedial hypothalamic nucleus
PVN: paraventricular nucleus
LHA: Lateral hypothalamic area
BST: bed nucleus of the stria terminalis
PVT: paraventricular nucleus of hypothalamus
GABA: gamma-aminobutyric acid
CO_2_: carbon dioxide
PSG: standard polysomnography
NREM: Non-Rapid eyes movement
ICV: intracerebroventricular
NEAT: non-exercise induced thermogenesis
SPA: spontaneous physical activity
LH: lateral hypothalamus
CNS: central nervous system
OP: obesity prone
OR: obesity resistant
PD: Parkinson's disease
VTA: ventral tegmental area
LTP: long-term potentiation
PTZ: Pentylenetetrazol
AT: orexin/ataxin-3-transgenic
BDNF: Brain-derived neurotrophic factor
VEGF: vascular endothelial growth factor
IGF-1: insuline-like growth factor.
